# Effects of Exhibit Text Presentation Types on Visual Attention Patterns and Cognitive Load in Digital Museums: An Eye-Tracking Study

**DOI:** 10.3390/jemr19040084

**Published:** 2026-08-03

**Authors:** Yingying Zhu, Ming Liu, Yinghuang Luo, Xuanjia Ren, Jinho Yim

**Affiliations:** 1 School of Art and Design, Beijing Institute of Fashion Technology, No. 2 East Yinghua Road, North End of Heping Street, Chaoyang District, Beijing 100029, China; 2 Department of Smart Experience Design, Graduate School of Techno Design, Kookmin University, Seoul 02707, Republic of Korea; 3 Department of Product Design, Academy of Fine Arts, Hanshan Normal University, Chaozhou 521021, China

**Keywords:** exhibit text presentation, eye tracking, cognitive load, reading behavior, visual attention patterns, digital museum

## Abstract

Exhibit texts are commonly presented as labels or information panels near artworks to provide visitors with direct access to key exhibit information. Digital museums offer flexible and diverse ways to present artworks and communicate their cultural meanings. They also allow exhibit texts to be displayed in different formats, which may shape users’ reading behavior and cognitive experience. This study used a repeated-measures, within-subject design (N = 38) to compare three types of exhibit text presentation: environment-based, object-based, and user-based. Eye tracking, the NASA Task Load Index, and semi-structured interviews were used to examine the users’ visual attention patterns and cognitive load. The results showed significant differences in cognitive load across presentation conditions (*p* < 0.05), with the object-based layout associated with relatively higher cognitive load and more active visual behavior. These findings provide empirical evidence on how exhibit text presentation affects visitors’ experience in digital museums and offer design implications for textual information in digital reading environments.

## 1. Introduction

With the development of digital technologies, art museums are no longer limited to physical spaces. Online platforms allow users to access temporal and geographic boundaries [1], expanding the dissemination and social impact of cultural heritage [2]. Digital museums provide richer forms of information presentation and new modes of cultural communication [3].

Studies have shown that information layout in interfaces affected the users’ visual attention patterns and information acquisition efficiency [4]. Users are more likely to be guided by interface layout than to browse randomly [5]. When related information is spatially proximate, users can integrate information more quickly and understand the content more effectively [6]. A well-organized information layout can improve the reading experience in digital environments [7].

In exhibition design, exhibit text is an important part of the overall exhibition narrative [8]. Text serves as a guide by supplementing exhibit content and helping users understand the creator’s ideas and the meaning of the exhibits [9]. Although information presentation has expanded to multimedia forms such as audio narration and animated demonstrations, texts remain one of the most familiar and widely accepted ways of communicating information to the public [10]. Existing studies on text in digital museums mainly focused on two aspects: interface-level presentation, such as text position, size, and interaction form, and content-level features, such as sentence structure, lexical complexity, and information density [11,12]. For example, Knaack found that enlarging floating text could shorten reading time and reduce visual discomfort [13]. J. Lee showed that medium-length text paragraphs provided the best readability and comprehension, whereas paragraphs that were too short or too long negatively affected reading performance [14]. Exhibit text design not only influences the users’ subjective comfort, but may also affect visual attention allocation during reading [15]. Previous studies have also discussed the relationships among interface layout, usability, user performance, and cognitive load [16]. In a study on website interface layout, Tang et al. found that layouts with matched text and images reduced older users’ cognitive load. Their findings also suggested that text served as the primary pathway for information retrieval [17].

Current research on digital museums mainly focuses on technical implementation, immersive experience, and interaction design [18,19,20]. In contrast, research on exhibit text information remains relatively limited. Existing text-related studies more often examine users’ needs for exhibit information, addressing the question of “what information should be provided”. However, the question of “how textual information should be presented” remains underexplored. Empirical research is still limited on whether different text presentation types affect users’ visual attention behavior and cognitive load. This study examines the role of different text presentation types in digital museums from the perspectives of reading behavior and cognitive experience. Research questions were as follows:

Q1: How do different exhibit text presentation types influence users’ visual attention patterns in digital museums?

Q2: How do different exhibit text presentation types influence users’ perceived cognitive load?

To answer these questions, we classified text presentation types into three categories: (a) environment-based, (b) object-based, and (c) user-based. We conducted a comparative experiment based on this classification. This study makes three main contributions. First, it focuses on exhibit text presentations in digital museums, a topic that has rarely been examined independently, and addresses the limited attention given to text design dimensions in existing research. Second, by combining eye-tracking data with subjective scale measures, this study analyzes the effects of different text presentation types from the perspectives of visual behavior and cognitive experience. Third, it provides preliminary empirical evidence for textual information design and user experience optimization in digital museums.

## 2. Related Works

### 2.1. Textual Information Design in Digital Museums

Text serves as a medium that connects visitors with exhibits. It plays an important role in shaping the museum experience [21]. Studies have shown that visitors reported greater enjoyment when exhibits were accompanied by text descriptions [22]. When reading museum texts, visitors may feel that “someone is talking to them”. The texts incorporate the thoughts of curators or collection owners and communicate exhibits more effectively. Effective museum texts need to balance scientific accuracy with audience comprehensibility [8]. In digital interfaces, texts not only need to convey content, but also need to adapt to different media. Taeha Yi et al. used eye tracking to explore users’ information needs across two media and found that visitors spent more time reading wall-based text information than information on mobile devices [4]. Novotny et al. found that text readability in three-dimensional virtual scenes was jointly influenced by font size, rendering conditions, and scene complexity [23].

### 2.2. Text Layouts in Digital Museums

In physical art museums, text is usually presented as fixed labels, whereas digital technologies expand the possibilities of text layout [24]. The Louvre Museum uses digital interactive guides in which text is organized through a separate digital interface and information is progressively presented according to reading depth [25]. The Acropolis Museum places textual information on handheld devices, allowing users to view interactive narratives related to physical exhibits through smartphones and tablets [26]. The framework proposed by T. Nunes used augmented reality (AR) to overlay digital text and graphical information onto cultural artifacts, providing additional artifact-related explanations [27]. These examples show that relationships among text, environment, objects, and users have changed. Several studies have discussed the presentation forms and positions of text in digital museums. Yao compared users’ perceptions of exhibit information dimensions under three text layouts [28]. The results showed that environment-based layouts were suitable for all information dimensions, whereas object-based layouts were more appropriate for presenting observable information. J. Wieland found that handheld devices were easier to use than head-mounted AR devices and provided a high quality user experience [29].

To categorize text panel layouts, this study uses the frame of reference as the basis for classification. In virtual environments, the choice of frame of reference determines how the position and orientation of an object or information are defined [30]. It affects users’ spatial understanding, perception, and interaction performance [31]. Based on the primary reference object to which the text is attached, three text presentation types in digital museums are identified: (1) environment-based layout, (2) object-based layout, and (3) user-based layout. Table 1 summarizes the main characteristics of different text layout types. The spatial contiguity principle in multimedia learning suggests that the spatial distance between textual and related visual information can influence users’ attention allocation [32,33]. Variations in the spatial relationships across these presentation types may be associated with differences in users’ visual attention patterns.

It should be noted that the text layouts discussed in this study are limited to textual information presented through the visual channel. This study focuses on the spatial position and presentation form of text in digital interfaces. Digital museums may also use multimodal forms such as audio narration, ambient sound, and haptic feedback. However, these factors introduce additional perceptual and cognitive variables, making it difficult to directly compare different text layouts. This study does not examine broader multimodal presentations involving auditory or haptic channels and limits the research scope to visual text design to improve the specificity and comparability of the experimental conditions.

### 2.3. Visual Attention and Eye-Tracking Technique

Eye-tracking technology can directly record behavioral features such as fixation location, fixation duration, and scan paths. These measures reflect users’ attention allocation patterns during visual tasks [36]. In museum research, eye tracking is used to reveal subtle patterns of users’ visual engagement, such as how they browse multimodal exhibitions and process exhibit information in real-time [37]. Previous eye-tracking studies have demonstrated that fixation-based measures, such as fixation count and fixation duration, provide important indicators of visual attention allocation [38]. Gaze transitions between different areas of interest (AOIs) have been used to examine attentional shifts and the integration of information across multiple representations [39]. Možina et al. showed that text format, font, and presentation medium changed the reading time, fixation behavior, and outcomes related to comprehension and memory [40]. Chen analyzed fixation count and dwell time to evaluate the effects of interface layout on users’ information search efficiency [41]. Changes in text design and information layout can alter users’ visual search strategies and attention allocation, which may further affect reading performance [42].

### 2.4. Cognitive Load Theory

Cognitive load theory (CLT) focuses on how information can be presented efficiently to reduce the load on working memory [43]. Studies showed that the split-attention effect became stronger as information integration difficulty and task complexity increased [44]. This theory has also been applied in digital museum research. It should be noted that visual attention and cognitive load represent related but distinct constructs. Eye-tracking measures primarily describe how users allocate visual attention, whereas cognitive load reflects users’ perceived mental effort during task performance. Information presentation affects users’ cognitive information comprehension experiences and perceived cognitive load [45]. Different interaction, organization, and task structure may result in varying levels of mental effort [46]. Pei et al. developed a multi-criteria decision-making framework to discuss the relationships among interface layout complexity, information density, brightness contrast, and cognitive load [20]. Karakus found that when the correspondence between text and exhibits or the surrounding environment was clearer, users could complete reading and comprehension with fewer cognitive resources [47]. Examining the effects of different text presentation types on users’ perceived cognitive load helps further explain differences in reading experience.

## 3. Materials and Methods

### 3.1. Materials

**Figure 1:**
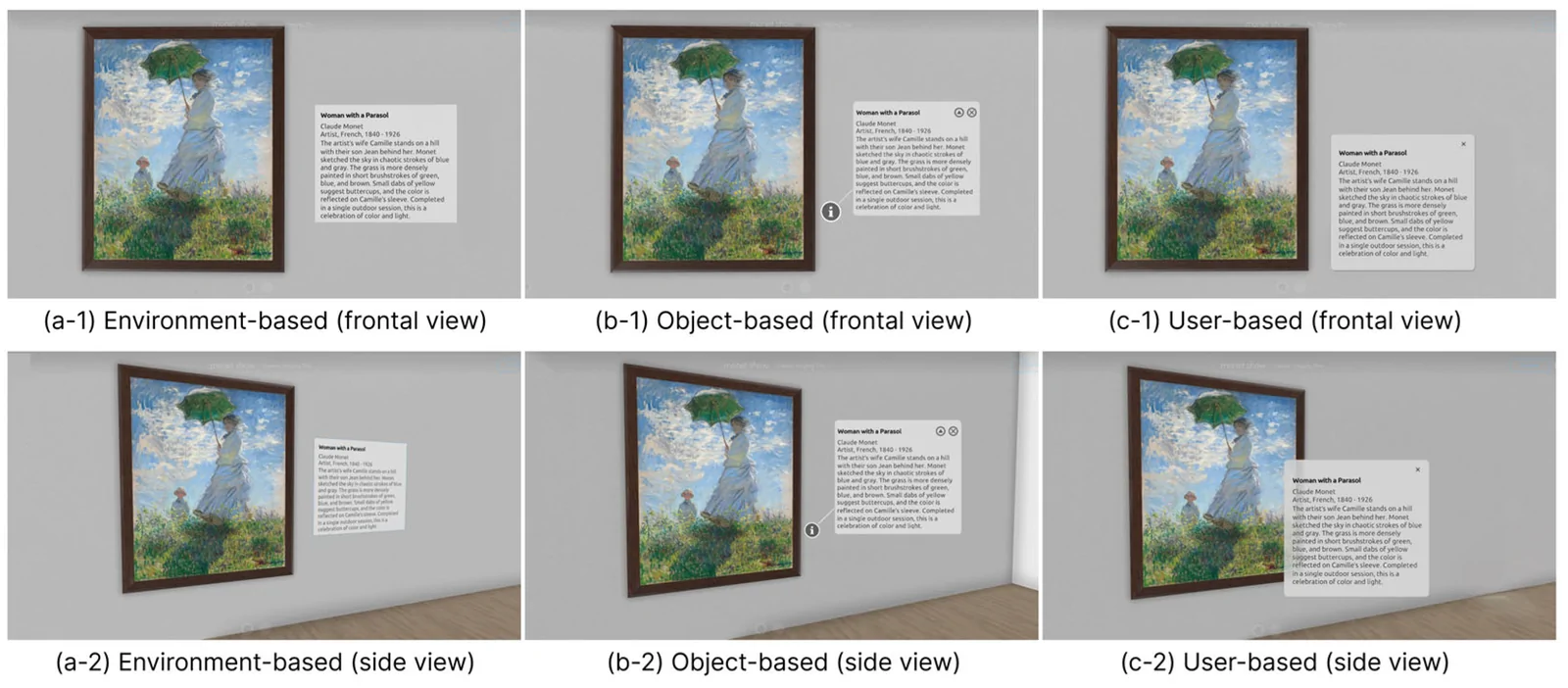
The figure shows the experimental stimuli used in the digital museum. (**a**-**1**,**a**-**2**) presents the environment-based condition; (**b**-**1**,**b**-**2**) presents the object-based condition; (**c**-**1**,**c**-**2**) presents the user-based condition. The upper row shows the frontal view, and the lower row shows the left-side view.

**Figure 2:**
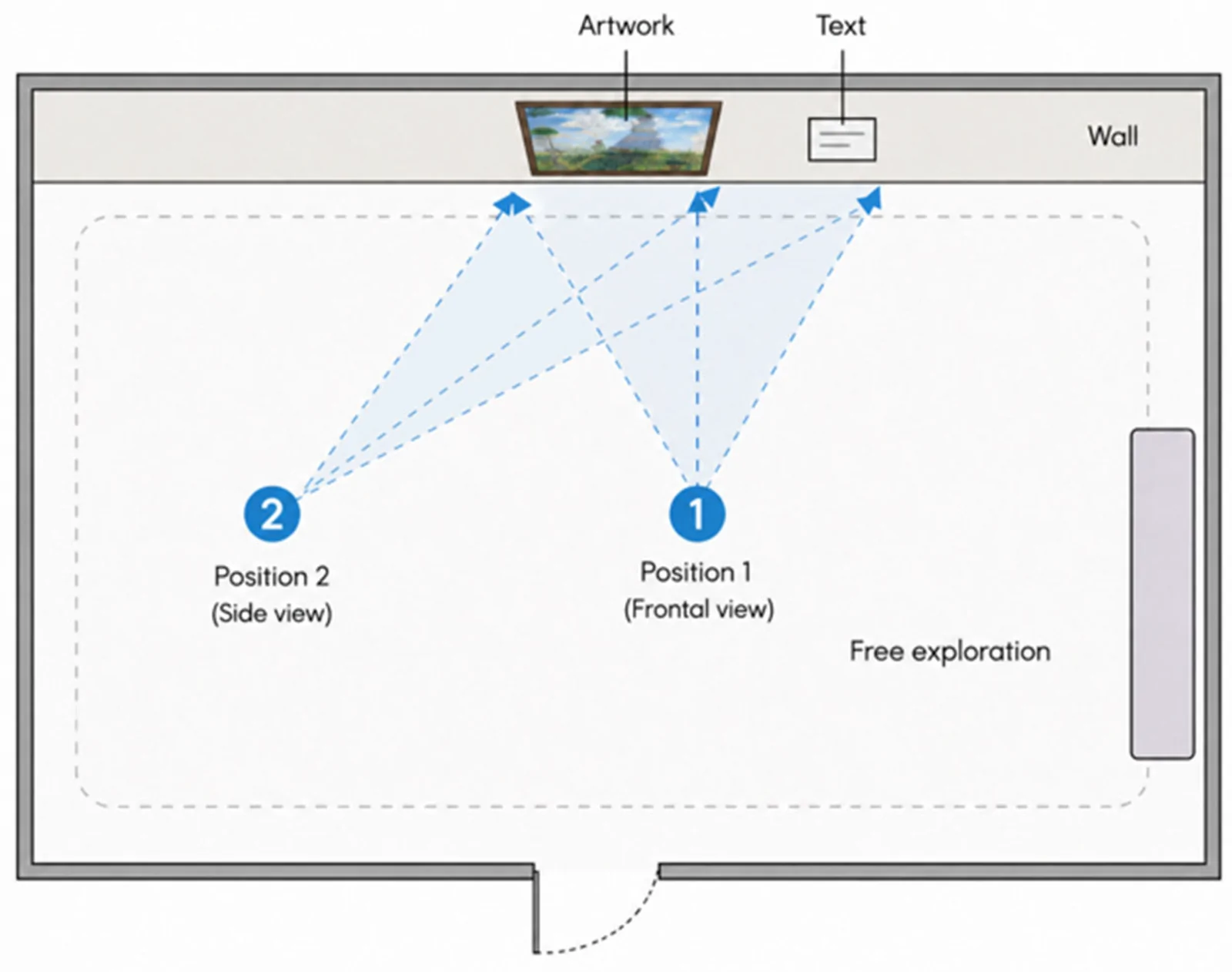
Schematic illustration of the predefined viewing positions and approximate viewing ranges in the experimental environment. Position 1 represents the frontal-view position, and Position 2 represents the side-view position. The shaded areas indicate the approximate fields of view covering both the artwork and the accompanying text.

To conduct this study, we created a digital art gallery exhibition. The exhibition included three scenes, and each scene contained one painting and its corresponding text description. The artworks were selected from the open-access digitized artwork resources of the National Gallery, which are released under the Creative Commons Zero (CC0) license. This study used the same artwork as the experimental stimulus to control for the potential effects of differences in visual complexity, subject matter, and artistic style. The text description of the painting included the artwork title, the artist’s name, the artwork overview, and an evaluative description. The text length was controlled at approximately 80 characters, based on previous studies on line length and readability [48]. The text was presented in a black Liberation Sans SDF font. All text was left-aligned, and the line spacing was kept relatively compact so that the information could be fully displayed within the text panel. File S1 presents the sample stimuli used in this study. The three text presentation types are shown in Figure 1. Each presentation type was examined from two predefined viewing positions: a frontal view and a left-side view, allowing the spatial appearance of the text to be observed from different angles. As illustrated in Figure 2, both predefined positions were arranged so that the artwork and its corresponding text remained within the participant’s approximate field of view. Position 1 represented the frontal-view position, whereas Position 2 represented the left-side-view position. In the subsequent data analysis, the eye-tracking metrics from the two viewing trials under the same text presentation condition were averaged.

(a) Environment-based (EN): The static text is embedded into the wall and fixed below the right side of the exhibit. When users move closer to or farther away from the exhibit, the text becomes larger or smaller according to the viewing distance.

(b) Object-based (OB): The text is attached to the exhibit and is displayed with the exhibit as the trigger point. The text panel adjusts its orientation according to the user’s viewing angle, such as by tilting vertically or rotating, so that it continues to face the user.

(c) User-based (US): The text is presented relative to the user’s viewpoint. It appears as an information panel similar to a head-up display and remains fixed on the right side of the screen. When the user moves or turns their head, the text moves synchronously with the user’s viewpoint and maintains a relatively fixed position within the field of view.

EN layout represents text with a static position and static orientation. OB represents text with a static position and dynamic orientation. US represents text with a dynamic position and static orientation.

### 3.2. Trial Setting

**Figure 3:**
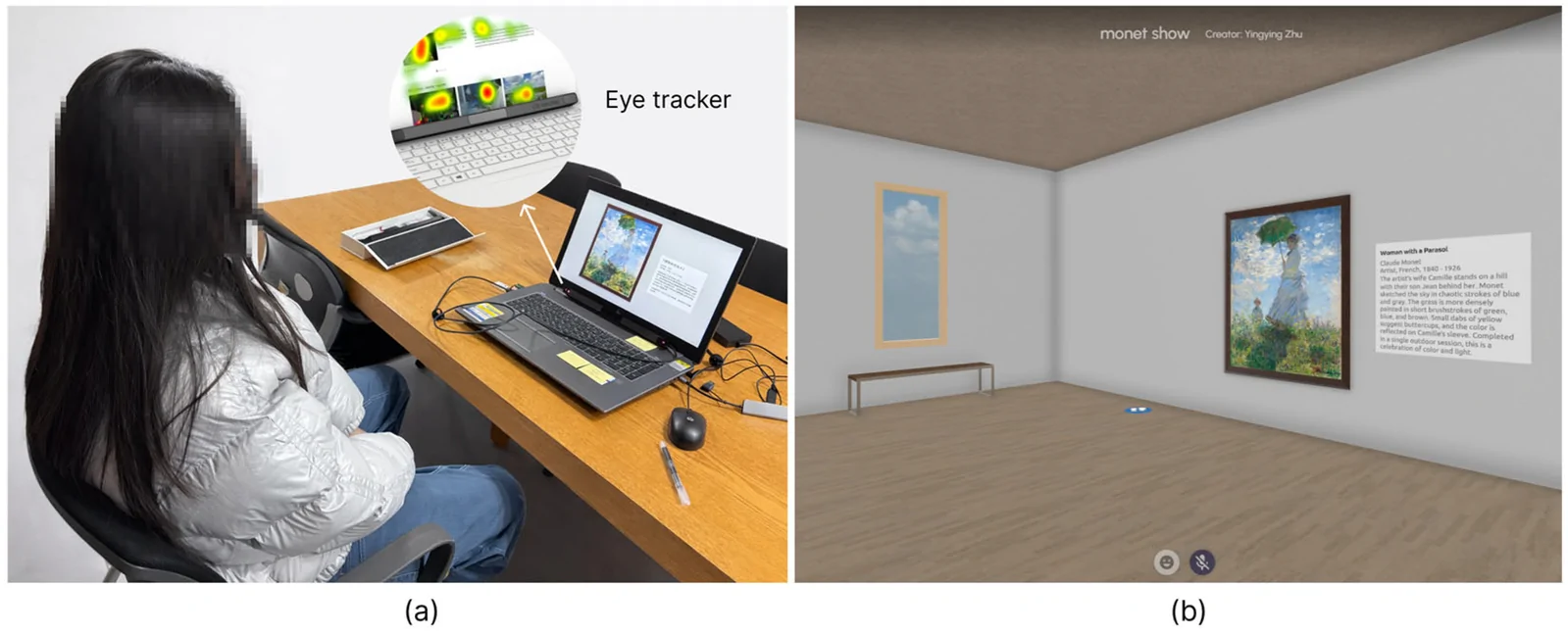
Experimental setup and digital museum environment. (**a**) Laboratory setup used for the eye-tracking experiment; (**b**) example of the digital environment used in the experiment, in which an artwork and its corresponding text panel were presented in a simulated exhibition space.

This study used the EyeLogic Lite (EyeLogic GmbH, Wintbach, Germany) eye-tracking system to record the participants’ eye-movement data. Figure 3 shows the experimental setup and an example of the digital environment used in the study. The device had a sampling rate of 120 Hz. The experimental stimuli were presented on a 15-inch screen with a resolution of 1920 × 1080 pixels. Participants were seated approximately 60–70 cm from the screen and were asked to maintain a stable sitting posture during the experiment. The experiment ran on a computer equipped with an Intel Core i5 processor and 16 GB of memory, using a 64-bit Windows operating system. The experiment was conducted in a university laboratory to ensure environmental consistency. The room was kept under stable indoor lighting conditions, and potential visual distractions and screen glare were minimized. Before the formal experiment, each participant completed a 9-point calibration to ensure the accuracy of the eye-tracking data. Calibration was repeated when the system indicated poor tracking quality.

### 3.3. Pilot Test

Before the formal experiment, we conducted a pilot test with six participants to evaluate the suitability of the experimental stimuli and procedure. The pilot test focused on the readability of the text panels, the clarity of the task instructions, the appropriateness of the experiment duration, and the stability of the eye-tracking calibration. Based on the pilot results, the wording of the task instructions was simplified to reduce ambiguity, and the calibration procedure was clarified to ensure more consistent eye-tracking quality. Data from the pilot test were not included in the final analysis.

### 3.4. Experimental Procedure

**Figure 4:**
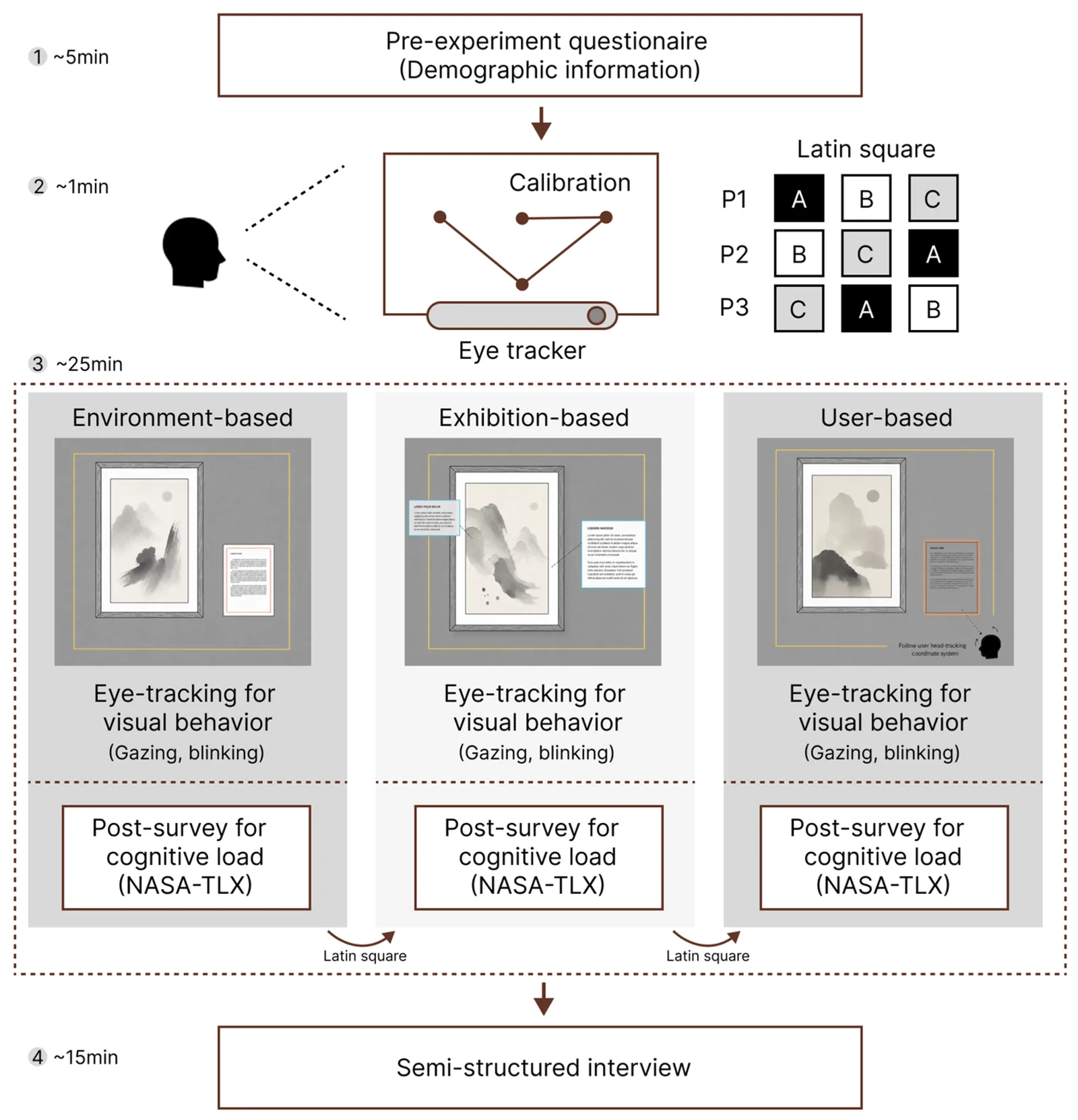
The experimental procedure and the use of a Latin square for the counterbalanced sequence of sessions.

This study adopted a four-stage mixed-methods design, as shown in Figure 4. In the first stage, participants completed a pre-experiment questionnaire. They provided basic demographic information, including gender, age, academic background, and prior experience with digital museums. The second stage involved experiment preparation and eye-tracking calibration. Before the formal experiment, the researchers explained the experimental task and procedure to the participants and calibrated the eye-tracking device to ensure the accuracy of data collection. To reduce the potential influence of presentation order across text presentation conditions, this study used a Latin square design to counterbalance the order of the three experimental conditions [49]. The three conditions were environment-based, object-based, and user-based text presentations. The third stage involved the formal experiment and immediate cognitive load assessment, which lasted approximately 25 min. For each condition, participants first entered the digital art gallery and moved to two pre-defined viewing positions: a front position and a side position. At each position, they were asked to view the artwork and read the accompanying text naturally. No fixed viewing time was imposed, and the participants proceeded when they felt that they had finished viewing and reading the exhibit information. Eye-movement data were recorded during these controlled viewing moments. The use of pre-defined viewing positions allowed the artwork and text panel to remain consistent in relative screen position and size across participants, supporting comparable eye-tracking measurements. After completing the two viewing trials for each condition, participants continued with a short free exploration task. This task allowed them to experience the spatial behavior and interaction characteristics of each text presentation type more naturally. Eye-tracking data collected during the free exploration phase were not included in the present analyses; only data recorded at the predefined front and side viewing positions contributed to the statistical analyses. After each condition, participants immediately completed the National Aeronautics and Space Administration Task Load Index (NASA-TLX) to evaluate their subjective cognitive load for that condition. Participants were allowed to take a short break between conditions to reduce fatigue. The fourth stage involved semi-structured interviews lasting approximately 10 to 20 min. The researchers interviewed the participants to further understand their subjective impressions, reading experiences, and reasons for their preferences regarding the three text presentation types. The interview data were used to supplement and explain the eye-tracking metrics and NASA-TLX results, providing a more comprehensive understanding of how different exhibit text presentation types affect users’ visual attention behavior and cognitive load.

To ensure consistency across participants and presentation conditions, the researcher followed a standardized operation guide during the experimental procedure, including instructions for entering the digital museum, viewing the artwork from predefined angles, completing the free exploration task, and filling out the NASA-TLX questionnaire (File S2).

### 3.5. Participants and Sample Size

A priori power analysis was conducted using G*Power 3.1 to estimate the required sample size. This study used a within-subject repeated-measures design. Parameter selection followed established conventions in behavioral and psychological research and the specifications of the present experimental design. The effect size was set at f = 0.25, representing a medium effect according to Cohen’s criteria and reflecting the expected differences in eye-tracking measures and subjective cognitive load among the three text presentation conditions [50]. The significance level was set at α = 0.05, statistical power was set at 0.80. Because directly comparable prior studies were limited, the conventional medium effect was adopted as a practical basis for sample-size estimation [51]. The number of measurements was set to 3, and the number of groups was set to 1. The calculation showed that the minimum required sample size was approximately 28 participants. To account for potential calibration failure and invalid or incomplete eye-tracking data, the target sample size was increased accordingly.

This study recruited 43 participants from a university in China. All participants had normal or corrected-to-normal vision. Participants were excluded if they had visually induced motion sickness, other conditions that could substantially affect cognitive function, or were taking medication that could influence cognitive performance. None had participated in a similar experiment or had prior knowledge of the experimental procedure. Participants were asked to avoid alcohol or any substances that could impair cognitive function within 24 h before the experiment. One participant failed to complete eye-tracking calibration, and four participants showed severe loss of gaze-tracking data. Five participants were excluded, and data from 38 participants were included in the final analysis. An additional sensitivity analysis was conducted using the same repeated-measures assumptions. With three measurements, an alpha level of 0.05, and a statistical power of 0.80, the study could detect a minimum within-subject effect size of f = 0.21. This estimate provides an approximate indication of the detectable condition effect.

The final sample included 20 women and 18 men, with a mean age of 28.18 years (SD = 3.93). Participants came from multiple disciplinary backgrounds, including design (n = 10, 26.3%), business (n = 9, 23.7%), sports (n = 8, 21.1%), music (n = 4, 10.5%), media (n = 3, 7.9%), science (n = 3, 7.9%), and dance (n = 1, 2.6%). Most participants had some experience browsing digital art. Seventeen participants reported using digital art platforms “occasionally” (44.74%), whereas only two reported having “never used” them (5.26%).

The Ethics Committee of a university in China approved this study. All participants signed an informed consent form before the experiment (File S3). The consent form covered participation in the eye-tracking experiment, subjective questionnaire assessment, and semi-structured interview. Participants were informed that they could stop the experiment and withdraw at any time if they experienced eye discomfort during the procedure. No participants withdrew during the experiment. After completing the experiment, each participant received a coffee voucher worth USD 4.

### 3.6. Measures

This experiment used a single-factor experimental design. The independent variable was the type of text presentation in the digital museum, and the dependent variables were users’ visual attention patterns and cognitive load. The study aimed to explore users’ behavioral characteristics under different text presentation conditions.

To answer Q1, which concerned visual attention patterns, we used an eye-tracking device to collect the participants’ visual behavior data. Areas of interest were defined for the analysis: the image area was defined as AOI 01, and the text area was defined as AOI 02. The analyzed eye-tracking metrics included blink rate, text AOI fixation count, text AOI fixation duration, text AOI mean fixation duration, and transition count between the image AOI and text AOI. Fixation-based measures were used to characterize participants’ visual attention allocation within the text area. AOI transition count was used to capture visual switching behavior and potential image-text integration between the image and text areas. Blink rate was included as a supplementary indicator related to visual fatigue and task engagement. The selection of these metrics was guided by the objective of examining visual attention allocation in digital museum environments. This study focused on multimodal information exploration rather than linear text reading processes. Therefore, reading-specific measures such as first fixation duration, regressions, and revisit counts were not included. The definition of each metric is provided in Table 2.

Eye-tracking data were processed using SMI BeGaze version 3.7.42 (SensoMotoric Instruments GmbH, Teltow, Germany). The software’s built-in low-speed event-detection procedure was applied. Fixations were identified using a dispersion-based algorithm. Saccades were defined as the intervals between adjacent fixation or blink events. Blinks were identified as periods of temporary loss of valid eye-position data. Samples with invalid gaze coordinates outside detected blink intervals were excluded from fixation and AOI-based calculations. After preprocessing, 727,282 out of 815,179 gaze samples were retained as valid, corresponding to an overall valid-sample rate of 89.2%. Condition-specific valid-sample rates ranged from 87.4% to 91.3%, indicating generally comparable tracking completeness across the three conditions.

To answer Q2, which concerned cognitive load, we used the NASA Task Load Index. NASA-TLX is a subjective cognitive load assessment scale that includes six dimensions. Mental Demand, Physical Demand, and Temporal Demand measure the demands placed on participants during the task. Effort, Performance, and Frustration focus on how participants respond to and manage the task [52]. NASA-TLX can reflect different levels of user experience and has been widely used in fields such as human–computer interaction, virtual reality, and psychophysiology [53,54]. It uses a 0–100 rating scale to assess subjective cognitive load after a task. Except for Performance, higher scores indicate higher load. Because the original Performance score indicates better perceived performance when the score is higher, this dimension was reverse coded before calculating the total score by subtracting the original Performance score from 100. After this transformation, higher scores across all dimensions indicate higher subjective cognitive load.

In addition, semi-structured interviews were conducted with participants to supplement the discussion of the two research questions. The questions were designed based on a three-level framework: (1) participants’ overall experience of digital museums; (2) their preferences regarding the three text presentation types from the perspectives of visual perception and information processing; and (3) their suggestions for improving future digital museums and text presentation formats. The semi-structured interview guide is provided in File S4. With the participants’ permission, the interviews were audio-recorded by the research staff.

### 3.7. Data Analysis

Data analysis was conducted to examine how different exhibit text presentation types influenced the users’ visual attention patterns and subjective cognitive load. Quantitative and qualitative analyses were performed to provide complementary perspectives on users’ behavioral responses and subjective experiences.

#### 3.7.1. Quantitative Data Analysis

Quantitative data were analyzed using IBM SPSS Statistics version 27.0 (IBM Corp., Armonk, NY, USA), with the significance level set at α = 0.05. Before the main analysis, supplementary linear mixed-effects analyses were conducted to assess two potential methodological influences: viewpoint and overall viewing time (File S5). For the viewpoint analysis, presentation condition, viewpoint, and their interaction were included as fixed effects, with participant included as a random intercept. No significant main effect of viewpoint was found across the principal eye-tracking measures. The front-view and side-view data were averaged to obtain an overall measure for each presentation condition. Given the self-paced nature of the task, overall viewing time was also examined as a potential confounding factor. Presentation condition had no significant effect on overall viewing time, indicating that the time spent completing the task was broadly comparable across the three conditions. Linear mixed models (LMMs) were used to examine the effects of text presentation condition on eye-tracking metrics and subjective cognitive load [55]. Although repeated-measures analysis of variance is commonly used for within-subject designs, LMMs provide a more flexible approach to account for repeated observations from the same participant [56]. Separate models were constructed for each eye-tracking metric and the NASA-TLX score. In each model, text presentation condition was included as a fixed effect, and participant was included as a random intercept. Alternative models including participant-specific random slopes for presentation condition were also evaluated. Because the addition of random slopes did not meaningfully improve model fit, the more parsimonious random-intercept structure was retained. The normality of model residuals was assessed using the Shapiro–Wilk test. No substantial deviations from normality were observed. When a significant main effect of condition was found, Bonferroni-corrected post hoc pairwise comparisons were performed. To quantify the magnitude of the condition effects, partial eta squared ($\eta_{p}^{2}$) was calculated from the F statistic and the corresponding numerator and denominator degrees of freedom. For significant post hoc pairwise comparisons, standardized effect sizes were approximated using Cohen’s *d* based on the model-derived contrast statistics. The absolute values of *d* were interpreted as small, medium, and large effects at approximately 0.20, 0.50, and 0.80. Model results were reported using F statistics, degrees of freedom, *p* values, partial eta squared values, estimated coefficients (β), standard errors (SEs), 95% confidence intervals, and Cohen’s *d* values.

#### 3.7.2. Qualitative Data Analysis

For qualitative data analysis, this study used NVivo to conduct thematic content analysis of the semi-structured interview data. Two researchers with experience in digital media informatics and museum exhibition design were involved in the analysis. The qualitative analysis consisted of three steps [57]. First, the two researchers independently reviewed the interview transcripts to become familiar with the data and research objectives before formal coding. During the initial familiarization stage, the researchers reviewed the transcripts while listening to the original interview recordings to verify transcript accuracy and gain a comprehensive understanding of the data. Second, an inductive coding approach was adopted to identify meaningful statements related to the participants’ reading experiences, information acquisition, visual comfort, and preferences. The two researchers independently coded the interview transcripts, and related codes were organized into higher-level categories and refined into themes. This process was repeated for each interview transcript. Third, after completing the analysis of all interview data, the two researchers compared the coding results. Disagreements regarding code assignment or theme interpretation were discussed and resolved through consensus. The qualitative findings were used to supplement and explain the eye-tracking metrics and NASA-TLX results, providing a more comprehensive understanding of how different text presentation types affected user experience.

## 4. Results

### 4.1. Quantitative Results

#### 4.1.1. Descriptive Statistics

The descriptive statistics are presented in Table 3. Overall, OB showed higher values than EN and US across several measures. Blink rate was highest in OB (M = 28.699 blinks/min, SD = 15.898, 95% CI [23.473, 33.924]), followed by US (M = 27.367 blinks/min, SD = 32.977, 95% CI [16.527, 38.206]) and EN (M = 18.815 blinks/min, SD = 12.491, 95% CI [14.709, 22.921]).

For text AOI-related measures, OB also showed relatively greater fixation engagement. Text AOI fixation count was highest in OB (M = 46.908, SD = 16.852, 95% CI [41.369, 52.447]), followed by EN (M = 41.118, SD = 16.943, 95% CI [35.549, 46.688]) and US (M = 37.947, SD = 17.019, 95% CI [32.353, 43.541]). Text AOI fixation duration was also highest in OB (M = 11.218 s, SD = 5.165, 95% CI [9.521, 12.916]), followed by EN (M = 9.465 s, SD = 4.569, 95% CI [7.964, 10.967]) and US (M = 8.936 s, SD = 4.634, 95% CI [7.412, 10.459]). The bidirectional transition count between the image AOI and the text AOI was also higher in OB (M = 9.592, SD = 5.115, 95% CI [7.911, 11.273]) than in EN (M = 7.026, SD = 4.278, 95% CI [5.620, 8.432]) and US (M = 7.592, SD = 5.902, 95% CI [5.652, 9.532]). In contrast, text AOI mean fixation duration varied only slightly across the three conditions, with EN showing the highest mean value (M = 239.592 ms, SD = 65.201, 95% CI [218.161, 261.023]).

For cognitive load, OB produced the highest NASA-TLX total score (M = 161.974, SD = 62.227, 95% CI [141.520, 182.427]), whereas EN and US showed lower values (EN: M = 108.105, SD = 67.014, 95% CI [86.078, 130.132]; US: M = 113.500, SD = 62.920, 95% CI [92.819, 134.181]). These descriptive results suggest that OB may be associated with greater visual engagement in the text area and higher subjective cognitive load.

**Figure 5:**
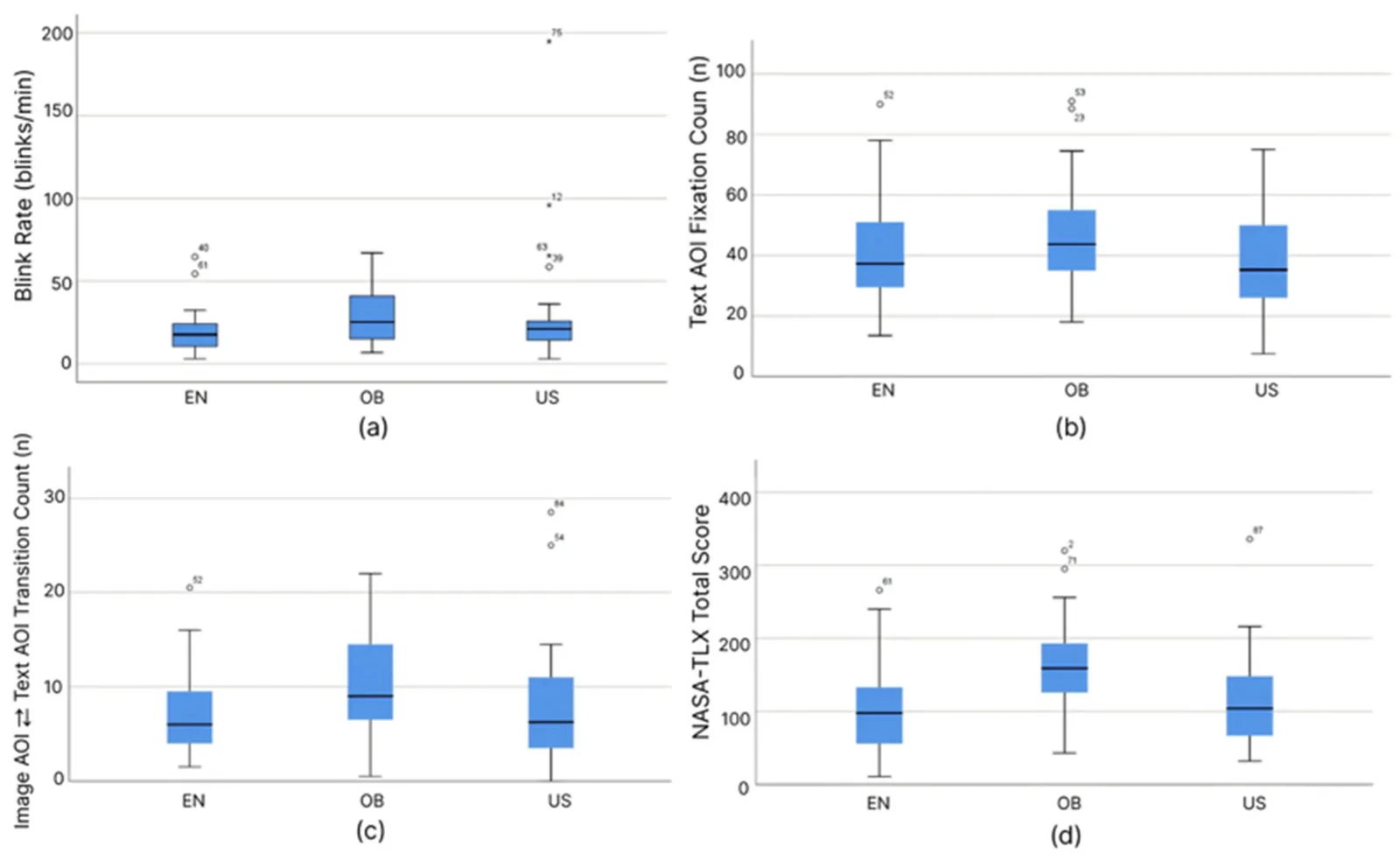
Boxplots of selected eye-tracking measures and cognitive load across text presentation conditions. The figure shows the distributions of four representative dependent measures: (**a**) blink rate, (**b**) text AOI fixation count, (**c**) image AOI ⇄ text AOI transition count, and (**d**) NASA-TLX total score. EN, OB, and US represent the environment-based, object-based, and user-based conditions, respectively. The horizontal line within each box represents the median, the box represents the interquartile range, the whiskers indicate the non-outlier range, circles indicate outliers, and asterisks indicate extreme outliers.

To provide an intuitive overview of the data distribution, boxplots were created for four representative measures: blink rate, text AOI fixation count, bidirectional transition count between the image AOI and the text AOI, and NASA-TLX total score (Figure 5). Overall, OB showed higher median values than EN and US, particularly for blink rate, text AOI fixation count, bidirectional transition count, and NASA-TLX total score.

#### 4.1.2. Linear Mixed-Effects Model Results

As shown in Table 4, the linear mixed model results indicated significant main effects of text presentation condition on several measures. For blink rate, the main effect of condition was significant (F(2, 74) = 3.485, *p* = 0.036, $\eta_{p}^{2}$ = 0.086). However, Bonferroni-corrected pairwise comparisons did not show significant differences between any two conditions. The difference between OB and EN approached significance (β = 9.884, SE = 4.063, 95% CI [−0.068, 19.837], *p* = 0.052); the differences between US and EN and between OB and US were not significant.

For AOI-related measures, conditions had a significant effect on text AOI fixation count (F(2, 74) = 4.846, *p* = 0.011, $\eta_{p}^{2}$ = 0.116). Post hoc comparisons showed that text AOI fixation count was significantly higher in OB than in US (β = 8.961, SE = 2.919, 95% CI [1.810, 16.111], *p* = 0.009, *d* = 0.70). Condition also had a significant effect on text AOI fixation duration (F(2, 74) = 4.266, *p* = 0.018, $\eta_{p}^{2}$ = 0.103). Post hoc comparisons showed that text AOI fixation duration was significantly higher in OB than in US (β = 2.283, SE = 0.818, 95% CI [0.279, 4.287], *p* = 0.020, *d* = 0.64). The effect of condition on text AOI mean fixation duration was not significant (F(2, 74) = 0.488, *p* = 0.616, $\eta_{p}^{2}$ = 0.013). This result indicates that different text presentation conditions did not significantly change the average duration of single fixations within the text area.

For the bidirectional transition count between the image AOI and the text AOI, the main effect of condition was significant (F(2, 74) = 3.858, *p* = 0.025, $\eta_{p}^{2}$ = 0.094). Bonferroni-corrected post hoc comparisons showed that the bidirectional transition count was significantly higher in OB than in EN (β = 2.566, SE = 0.971, 95% CI [0.188, 4.943], *p* = 0.030, *d* = 0.61). This result indicates that participants made more frequent gaze shifts between the image and the text in the OB condition.

Text presentation condition had a significant main effect on NASA-TLX total score (F(2, 74) = 13.106, *p* < 0.001, $\eta_{p}^{2}$ = 0.262). Post hoc comparisons showed that cognitive load was significantly higher in OB than in EN (β = 53.868, SE = 11.589, 95% CI [25.480, 82.257], *p* < 0.001, *d* = 1.07). It was also significantly higher in OB than in US (β = 48.474, SE = 11.589, 95% CI [20.085, 76.862], *p* < 0.001, *d* = 0.96). These results indicate that OB induced the highest subjective cognitive load among the three text presentation types.

Overall, the linear mixed model results showed that the object-based condition was associated with greater allocation of visual attention and cognitive load across several measures. The object-based condition showed higher text AOI fixation count and total fixation duration, more frequent gaze transitions between the image AOI and the text AOI, and a higher NASA-TLX total score. However, text AOI mean fixation duration was not significantly affected by the text presentation condition. These findings suggest that different text presentation types influenced the participants’ fixation allocation, gaze transitions, and subjective cognitive load.

### 4.2. Qualitative Feedback

To supplement the eye-tracking data and NASA-TLX results, this study conducted a thematic analysis of semi-structured interviews. The interview results showed that participants’ evaluations of the three text presentation types mainly focused on four aspects: intuitiveness and familiarity, image-text integration and attention switching, sense of control and perceived distraction, and future needs for information presentation. The following sections present representative views and thematic elements under each theme.

#### 4.2.1. Theme 1: Intuitiveness and Familiarity

The first theme was intuitiveness and familiarity. Many participants considered the environment-based layout to be the easiest to understand. For example, Participant 21 explicitly stated a preference for a “simple and intuitive” presentation. One participant noted, “Because it is similar to the label style in traditional museums, with text descriptions placed directly below or beside the painting, I do not need to click anything extra” (P26). Some participants felt that the user-based layout resembled mobile or web interfaces and provided a stronger sense of familiarity with digital reading (P24). These responses suggest that the readability of text presentation depends not only on its similarity to traditional exhibition experiences, but also on whether it aligns with users’ habits of using digital interfaces.

#### 4.2.2. Theme 2: Image-Text Integration and Attention Switching

The second theme was image-text integration and attention switching. Participants reported that text placed close to the artwork helped them understand the work more easily. One participant stated, “The text and the image formed a naturally coordinated viewing angle, which reduced eye movement and did not interrupt my thinking process. Therefore, it felt the most comfortable to read” (P28). Participant 7 also noted that the object-based layout was flexible. Another group of participants felt that the environment-based layout supported understanding through the juxtaposition of image and text within the same space. One participant noted that in the environment-based layout, the text and artwork were in the same spatial context without a clear boundary, making it easier to build associations and understand the background of the work (P32). This suggests that the environment-based layout supports image–text integration in a way that is closer to the traditional “artwork-label” juxtaposition in physical exhibitions, whereas the object-based layout emphasizes a more dynamic relationship between text and object. However, this advantage was also accompanied by more frequent attention switching. Participants needed to continuously coordinate their attention among the artwork, the text panel, and changes in viewing angle (P6). The object-based layout may both promote image-text integration and increase the need for visual attention switching between the image and the text.

#### 4.2.3. Theme 3: Sense of Control and Perceived Distraction

The third theme was sense of control and perceived distraction. The interviews showed that the participants did not necessarily reject pop-up text. When the text was actively triggered by the user and could be closed when needed, participants generally did not perceive it as interrupting the viewing experience (P22, P37). In contrast, participants were more likely to feel distracted when the text was always visible or when its pop-up position and trigger mechanism were unclear. One participant stated, “I can first view the artwork and then choose whether to read the description” (P15). In the environment-based layout, the text was always visible. Although this made the presentation intuitive, users could not hide the information. The object-based and the user-based layouts provided a stronger sense of control through click-based or pop-up presentation. Text presentations in digital museums should not only consider whether the text is visible, but also whether users can control when the text appears, where it appears, and how deeply they want to read.

#### 4.2.4. Theme 4: Future Needs for Information Presentation

The fourth theme was future needs for information presentation. Participants generally believed that text descriptions in digital museums should not simply replicate labels from physical exhibitions. They should make better use of the affordances of digital media. Regarding text optimization, participants suggested using a layered structure (P28). One participant stated, “The text could first provide concise basic information and then allow users to click to expand deeper information, such as the artist’s background, the creative context, related works, or historical context” (P29). These responses indicate that future information design in digital exhibitions should balance the concise presentation of basic information with expandable access to more in-depth content. In addition, participants suggested AI-based question-answering or knowledge-linking functions (P06), indicating that users expect digital exhibitions to provide more personalized and interactive ways of accessing information.

## 5. Discussion

### 5.1. Principal Results

This study examined how three exhibit text presentation types were associated with users’ visual attention behavior and cognitive load in a digital museum. The quantitative results showed that the three text presentations produced different patterns of visual attention allocation and subjective cognitive load. The object-based condition showed greater visual engagement with the text across several eye-tracking metrics, including text AOI fixation count and text AOI fixation duration. The NASA-TLX total score was also higher in the object-based conditions than in the environment-based and user-based conditions. These results suggest that although object-based layout may draw stronger attention to the text and support comparison between the artwork and the text, it may also impose higher subjective cognitive load.

This finding responds to the two research questions proposed in this study. For Q1, the results showed that different exhibit text presentation types led to different visual attention patterns, particularly in terms of fixation engagement within the text area and gaze transitions between the artwork and the text. For Q2, the results showed that text presentation type also affected subjective cognitive load. The object-based layout produced the highest cognitive load, whereas the environment-based and the user-based layout produced relatively lower cognitive loads.

### 5.2. Interpretation of the Findings

#### 5.2.1. Environment-Based Layout

The environment-based layout fixed the text within the virtual environment, like wall labels or information panels. The interview results showed that many participants considered the environment-based layout the easiest to understand because it matched their existing experience of physical museums: text descriptions are placed near the artwork, and users can read them when they move closer [9]. This low operational cost may explain why subjective cognitive load was relatively lower in the environment-based condition. The advantage of the environment-based layout is that it preserves the familiar “artwork-label” relationship found in physical exhibitions, allowing users to complete the reading task with a lower learning cost [34]. Because the text is fixed in the environment, its readability may be reduced by perspective distortion when users view it from an oblique angle [58]. If users need to adjust their viewpoint to clearly see both the artwork and the text, this may also increase the local cost of visual search [59].

In this experiment, participants showed the fewest gaze transitions between the image and the text in the environment-based layout condition, while their mean fixation duration on the text AOI was the longest. This pattern may indicate that users formed relatively longer gazes within the text area. Reitstätter et al. showed that visitors’ reading patterns included “quick looks” and “long gazes”, which serve different functions [60]. Long gazes transform symbolic information in the world into knowledge and represent a form of memorable look [61]. This characteristic suggests that the environment-based layout may be more suitable for basic information, short texts, and stable explanatory content. When the text is brief and clearly structured, the longer gazes formed in the environment-based layout may support sustained processing of exhibit information [62].

#### 5.2.2. Object-Based Layout

The object-based layout attached the text near the exhibit and maintained an appropriate orientation according to the user’s viewing angle. Some participants felt that this layout connected the text description with the artwork more naturally, and they tended to view the textual information more frequently. The quantitative results appeared to reflect this viewing behavior. This feedback was consistent with the higher text AOI fixation count, longer cumulative fixation duration, and more frequent image–text transitions. These findings indicate that participants allocated more visual attention to the textual information and shifted their gaze more frequently between the text and artwork under the object-based condition. Such behavior may be associated with more extensive information processing activity, but it may also partly reflect additional information search or spatial navigation demands [63,64]. The present findings are primarily interpreted as evidence of a more active and dynamic pattern of visual exploration rather than as direct evidence of deeper processing or improved comprehension. A study on reading in virtual reality showed that object-based layout types can help create a dynamic experience, making users feel surrounded by information [28].

Participants showed a higher NASA-TLX total score in the object-based layout condition than in the other two conditions. One possible interpretation is that when users need to coordinate multiple information sources, their working memory demands may increase [65]. In addition, the higher subjective workload may reflect the greater visual navigation demands of the object-based condition [66]. As the text moved relative to the artwork, users may have needed to redirect their visual attention to maintain the spatial relationship between the two.

The object-based layout was associated with more frequent visual coordination between textual and visual information and a more active pattern of visual exploration. This layout may also increase visual navigation demands and subjective task workload. This finding is broadly consistent with Makransky et al., who found that higher immersion and richer visual information could increase cognitive load and attention distraction [67]. The object-based layout may be more suitable for explaining local details or highlighting key information about an exhibit, as it can help users map textual content onto corresponding visual elements.

#### 5.2.3. User-Based Layout

The user-based layout fixed the text at a relative position within the user’s field of view, like a webpage, mobile interface, or head-up display information panel. For users who are accustomed to reading on mobile devices, websites, or software interfaces, the user-based layout may provide a strong sense of familiarity with digital interfaces [68]. Some participants felt that the user-based layout was more stable to read because the text position was not affected by wall perspective or the user’s standing position. Rzayev found that a head-fixed position allowed participants to read text without needing to move closer to it [34]. However, some participants felt that the user-based layout was somewhat separated from the digital environment. When the text appeared too much like an independent user interface, visitors felt that they were reading a webpage rather than participating in an integrated digital museum experience [69].

For longer background descriptions, artist information, or expandable layers of in-depth content, the user-based layout may be more suitable as an auxiliary information panel. However, for content that requires visitors to carefully compare local details of the artwork with textual explanations, a strong interface-like presentation may weaken image-text integration and spatial immersion [70].

### 5.3. Relationship to Previous Studies

Some of the present findings are consistent with previous studies. Visitors’ viewing patterns became more complex and diverse, shifting beyond the basic “artwork–label–artwork” pattern [60]. This is consistent with the present findings, which showed that different text presentation types produced significant differences in gaze transitions between the artwork and the text. Significant differences were found in text AOI fixation count and fixation duration, whereas mean fixation duration remained comparable across conditions. A study in an AR environment found that total fixation duration, fixation frequency, and mean fixation duration showed different sensitivities to different task conditions [71]. In the present study, the text presentation types primarily influenced how frequently users attended to the text and the amount of cumulative visual attention allocated to the text AOI. The duration of individual fixations remained relatively stable across conditions [72]. The object-based layout produced higher cognitive load, which also echoes previous research on information-rich environments. Immersive virtual reality can enhance presence, but it may also create challenges for learning [73]. Stronger spatial connection and richer visual information may support image-text integration, but they may also increase the need for users to switch attention among the artwork, the text, and interface changes [74]. Frequent attention switching may introduce search and orientation processes that are not directly related to the learning goal itself, thereby adding potential distraction [75]. This finding prompts further reflection on text presentation design. Like the design of learning materials in educational contexts, digital museums also need optimized design strategies to reduce extraneous cognitive load and help users coordinate exhibit and textual information more effectively. For blink rate, although the overall model indicated a significant effect of presentation condition, none of the pairwise comparisons remained significant after Bonferroni correction. This finding should be interpreted cautiously. Some studies reported that blink rate decreased when cognitive load and visual attention demand increased, whereas others found that blink rate increased with greater task demands [76,77]. Blink rate should not be treated as a single linear indicator of cognitive load. It may reflect a combination of task characteristics, sustained visual attention, and physiological regulation [78].

### 5.4. Limitations

This study has several limitations. First, the participants were mainly young adults with some experience using digital devices. The participant sample was relatively homogeneous, and participants’ familiarity with digital interfaces may have influenced their reading behavior and perceived cognitive load. Future studies should include a larger and more diverse sample, such as older adults and children, to examine differences among user groups in their preferences for text layouts and perceived cognitive load. Second, the participants were recruited from a limited geographic area. Museum visiting experience and preferences for reading art-related information may be influenced by local educational backgrounds and cultural contexts. The generalization of the findings to other countries, regions, or cultural settings should be treated with caution. Third, this study used a controlled digital museum environment and specific exhibit texts. To control experimental variables, the artwork and text content were kept consistent across the three conditions. This approach helped improve internal validity but also limited the external generalizability of the findings. A single artwork may not fully reflect differences in visual attention and cognitive load across artistic styles and levels of visual complexity. In addition, the predefined viewing positions and relatively simplified setting may not fully reflect visitors’ natural exploratory behavior in real digital museums. Future studies could include multiple works representing different art forms, such as oil paintings, sculptures, and digital art, in existing digital museums. They should also consider how spatial configuration and the presentation of multiple exhibits within the same scene affect visitors’ reading behavior, cognitive load, and expectations regarding text layout. Fourth, the range of eye-tracking metrics included in this study was relatively limited. More consistent differences across conditions were observed in text AOI fixation count, text AOI fixation duration, image–text transition count. In contrast, blink rate may also be influenced by physiological, environmental, and task-related factors. The corresponding findings should be interpreted with caution. Future research could incorporate additional measures, such as pupil dilation, gaze entropy, and saccade characteristics, to broaden the assessment of users’ visual attention and cognitive responses. As an exploratory study, this work aimed to capture visitors’ visual attention allocation, reading behavior, and subjective task load rather than directly assessing learning outcomes. Future research should incorporate task-performance measures, such as memory for artwork information, comprehension accuracy, and reading completion time, to further examine the relationships among visual attention, subjective workload, and learning performance.

## 6. Conclusions

This study examined how three exhibit text presentation types in digital museums—environment-based layout, object-based layout, and user-based layout—affect users’ visual attention patterns and cognitive load. The main contribution of this study focused on how exhibit text can be positioned and presented within virtual exhibition spaces. By combining eye-tracking data, NASA-TLX results, and semi-structured interviews, this study provided a multidimensional understanding of users’ attention allocation and reading preferences under different text presentation conditions. The results showed that the object-based layout was associated with greater visual attention to the text and more frequent gaze transitions between the artwork and textual information while also producing higher subjective task workload. The environment-based layout was recognized for its familiarity and intuitiveness, whereas the user-based layout supported more stable and efficient reading but could also make users feel that the text was somewhat separated. Text presentation in digital museums is not only a matter of readability; it also appears to be a design factor that influences visual attention, image–text integration, and subjective cognitive effort. By examining the effects of different text presentation types from the perspectives of cognitive experience and visual behavior, this study provides preliminary evidence for understanding the relationships among text layout, visual attention allocation, and cognitive load in digital environments. These findings offer useful references for designers and developers of museums and provide new insights into the design of interactive systems that present textual information in spatial digital environments.

## Figures and Tables

**Table 1:** Layout types of text based on the frame of reference.

Layout Type	Text Presentation	Characteristics	References
Environment-based	Wall panel World-fixed	The text is fixed at an absolute position, such as on a wall. Regardless of how the user moves or changes viewing direction, the text does not move with the user’s viewpoint.	Y. Yao [28] R. Rufat [34]
Object-based	Above the artifact Edge-fixed Side of the artifact Floating label	Text is attached to the exhibit and remains near the object during user movement. The text window is typically displayed near the object, such as in front of or above it.	Y. Yao [28] R. Rufat [34] Y. Wang [35]
User-based	Head-fixed Hand-fixed Controller display	Text is fixed within the user-centered coordinate system and moves with the user’s head or controller. It maintains a stable position relative to the user’s viewpoint.	R. Rufat [34] J. Lee [14] G. Lee [7]

**Table 2:** Eye-tracking metrics and operational definitions.

Indicator	Meaning
Blink rate	The number of blinks occurring per unit of time, usually expressed as blinks per minute.
Text AOI fixation count *	The number of fixations made by participants within the text AOI.
Text AOI fixation duration	The sum of the duration of all fixations within the text AOI during one experimental trial.
Text AOI Mean Fixation duration	Text AOI total fixation duration divided by text AOI fixation count, representing the average duration of each fixation within the text area.
Image AOI ↔ Text AOI transition count	The total number of bidirectional gaze transitions between AOI 01 (image) and AOI 02 (text), including transitions from the Image AOI to the text AOI and from the text AOI to the Image AOI.

* AOI = area of interest.

**Table 3:** Descriptive statistics of eye-tracking measures and cognitive load across the three text presentation conditions.

	EN *		OB *		US *	
Measure	Mean ± SD	95% CI	Mean ± SD	95% CI	Mean ± SD	95% CI
Blink Rate, blinks/min	18.815 ± 12.491	14.709–22.921	28.699 ± 15.898	23.473–33.924	27.367 ± 32.977	16.527–38.206
Text AOI Fixation Count, n	41.118 ± 16.943	35.549–46.688	46.908 ± 16.852	41.369–52.447	37.947 ± 17.019	32.353–43.541
Text AOI Fixation Duration, s	9.465 ± 4.569	7.964–10.967	11.218 ± 5.165	9.521–12.916	8.936 ± 4.634	7.412–10.459
Text AOI Mean Fixation Duration, ms	239.592 ± 65.201	218.161–261.023	229.674 ± 60.373	209.830–249.518	232.739 ± 60.832	212.744–252.735
Image AOI ⇄ Text AOI Transition Count, n	7.026 ± 4.278	5.620–8.432	9.592 ± 5.115	7.911–11.273	7.592 ± 5.902	5.652–9.532
NASA-TLX Total Scores	108.105 ± 67.014	86.078–130.132	161.974 ± 62.227	141.520–182.427	113.500 ± 62.920	92.819–134.181

* EN = environment-based condition; OB = object-based condition; US = user-based condition. Values are reported as mean and standard deviation. Fixation duration is reported in seconds; mean fixation duration is reported in milliseconds. AOI = area of interest; NASA-TLX = National Aeronautics and Space Administration Task Load Index.

**Table 4:** Linear mixed-effects model results for the effect of text presentation condition on eye-tracking measures and cognitive load.

Outcome	df1	df2	F	*p* Value	Partial $\eta_{p}^{2}$
Blink rate	2	74	3.485	0.036	0.086
Text AOI fixation count *	2	74	4.846	0.011	0.116
Text AOI fixation duration	2	74	4.266	0.018	0.103
Text AOI mean fixation duration	2	74	0.488	0.616	0.013
Image AOI ⇄ text AOI transition count	2	74	3.858	0.025	0.094
NASA-TLX total scores *	2	74	13.106	<0.001	0.262

* AOI = area of interest; NASA-TLX = National Aeronautics and Space Administration Task Load Index. Partial eta squared ($\eta_{p}^{2}$) was calculated from the F statistic and the corresponding numerator and denominator degrees of freedom.

## Data Availability

Deidentified data supporting the findings of this study are available from the corresponding author upon reasonable request, subject to ethical approval and compliance with the terms of participant consent. The data are not publicly available due to privacy and ethical restrictions related to human participant data.

## Supplementary Materials

The following supporting information can be downloaded at: https://www.mdpi.com/article/10.3390/jemr19040084/s1, File S1: Artwork information used in the experimental stimuli; File S2: Standardized participant instructions for the digital museum exploration task; File S3: Interview consent form and participant rights statement; File S4: Semi-structured interview protocol based on a three-level framework; File S5: Supplementary analysis of viewpoint and overall viewing time effects on eye-tracking measures.

## Abbreviations

The following abbreviations are used in this manuscript:

AR	Augmented Reality
AOI	Areas of Interest
LMM	Linear Mixed Model
NASA-TLX	National Aeronautics and Space Administration Task Load Index
