# Supplementary material for: Effects of Exhibit Text Presentation Types on Visual Attention Patterns and Cognitive Load in Digital Museums: An Eye-Tracking Study
Source: J Eye Mov Res. 2026 Aug 3;19(4):84. doi: 10.3390/jemr19040084 (PMC13514559; doi:10.3390/jemr19040084)
Supplement: Supplementary file 1 [file jemr-19-00084-s001.zip › File S1.pdf]

File S1: Artwork Information Used in the Experimental Stimuli

Artwork Title: Woman with a Parasol - Madame Monet and Her Son
Artist Name: Claude Monet
Occupation, Place of Birth, Date of Birth: Artist, French, 1840 - 1926
Artwork Overview and Evaluation: The artist's wife Camille stands on a hill with their son Jean behind her. Monet sketched the sky in chaotic strokes of blue and gray. The grass is more densely painted in short brushstrokes of green, blue, and brown. Small dabs of yellow suggest buttercups, and the color is reflected on Camille's sleeve. Completed in a single outdoor session, this is a celebration of color and light.

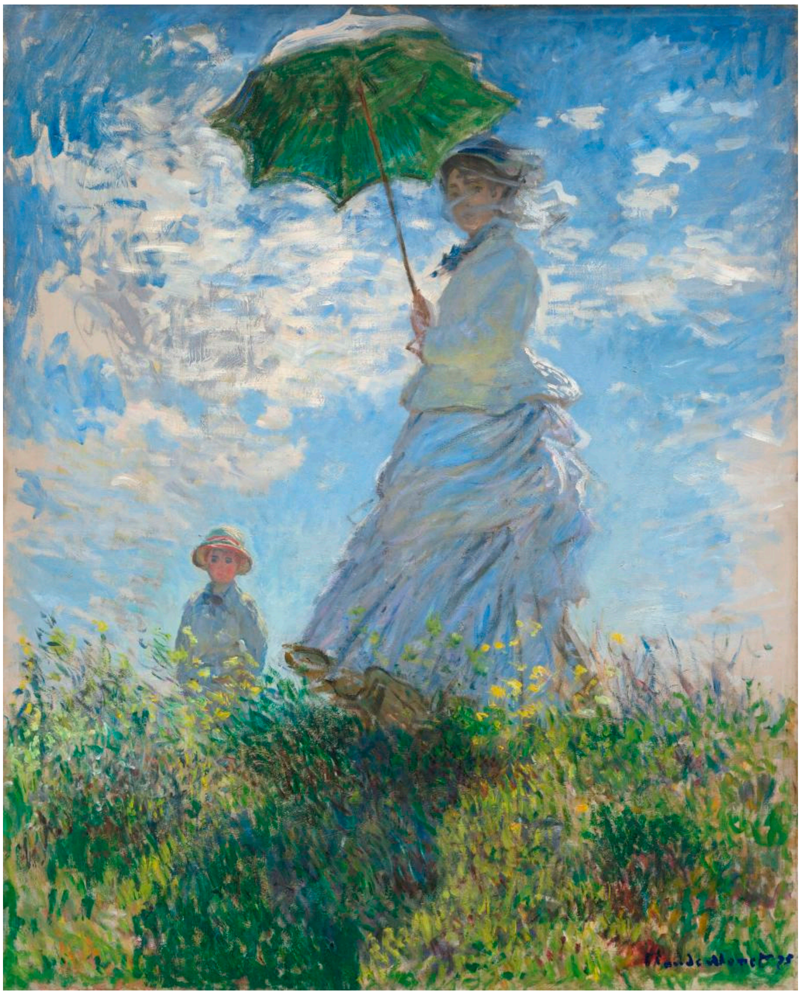

Image source: National Gallery of Art

https://www.nga.gov/artworks/61379-woman-parasol-madame-monet-and-her-son
