# Supplementary material for: Effects of Exhibit Text Presentation Types on Visual Attention Patterns and Cognitive Load in Digital Museums: An Eye-Tracking Study
Source: J Eye Mov Res. 2026 Aug 3;19(4):84. doi: 10.3390/jemr19040084 (PMC13514559; doi:10.3390/jemr19040084)
Supplement: Supplementary file 1 [file jemr-19-00084-s001.zip › File S2.pdf]

File S2 . Participant Instructions for the Exploration Task

Step	Experimental Phase	Instruction Given to Participants
1	Entering the digital art museum	“Please open the door in front of you and enter the museum.”
2	Orientation adjustment	“After entering the museum, please adjust your view to face the main exhibition area. You may use the mouse, touchpad, or controller to adjust your viewing direction.”
3	Familiarization with movement	“Please try moving around in the space and briefly observe the surrounding exhibits.”
4	Moving to the artwork	“Please move toward the artwork: the leftmost / middle / rightmost painting in the exhibition area, and stop in front of the artwork.”
5	Viewing from the first predefined angle	“Please view the artwork and read the accompanying text from this position. You may adjust your viewing direction slightly if needed. Please tell the researcher when you have finished reading.”
6	Viewing from the second predefined angle	“Now please view the same artwork and text from another viewing angle. Please read the text naturally and tell the researcher when you have finished reading.”
7	Free exploration	“You may now continue to explore this exhibit freely. You can move closer to or farther away from the artwork, adjust your viewing angle, and observe how the text is presented in relation to the artwork.”
8	Completion of the condition	“When you feel that you have finished viewing and reading this exhibit, please tell the researcher, ‘I have finished.’”
9	NASA-TLX questionnaire	“Please complete the corresponding section of the questionnaire based on your experience with this text presentation condition.”
10	Repetition for the next condition	“Next, please move to the artwork: the leftmost / middle / rightmost painting. Please repeat the same procedure: viewing from the predefined angles, free exploration, and questionnaire completion.”
11	End of the experiment	“Thank you. You have completed all experimental tasks. Please submit the questionnaire.”

Researcher notes:

The researcher recorded the start and end points of each predefined viewing trial. Eye-tracking data were collected during the two predefined viewing trials for each text presentation condition. The subsequent free exploration task was used to support participants’ natural experience of the text presentation type before completing the NASA-TLX questionnaire.
