# Supplementary material for: Effects of Exhibit Text Presentation Types on Visual Attention Patterns and Cognitive Load in Digital Museums: An Eye-Tracking Study
Source: J Eye Mov Res. 2026 Aug 3;19(4):84. doi: 10.3390/jemr19040084 (PMC13514559; doi:10.3390/jemr19040084)
Supplement: Supplementary file 1 [file jemr-19-00084-s001.zip › File S3.pdf]

INTERVIEW CONSENT FORM

Study Title: Effects of Exhibit Text Presentation Types on Visual Attention Patterns and Cognitive Load in Digital Museums

Understanding of my rights in research

Please circle YES or NO

I read the letter about this study.	**YES**	**NO**
I decided that I want to be in this study.	**YES**	**NO**
I know that I do not need to be in this study if I do not want to.	**YES**	**NO**
I will participate in this study. The seesion will last 40-50 minutes.	**YES**	**NO**
I can stop my participation when I want.	**YES**	**NO**
It is okay for the researchers to use my answers when they tell people about their research.	**YES**	**NO**
I know that the researchers will not tell anybody my name.	**YES**	**NO**
It is okay for the researchers to ask me again if I want to continue participating in the study.	**YES**	**NO**
I understand that my eye movements may be recorded during the study.	**YES**	**NO**
I know that my personal information will be kept confidential.	**YES**	**NO**

Participants:

I had an opportunity to discuss this study, and any questions that I have asked were answered to my satisfaction. I voluntarily consent to participate in the **interview** and **subjective questionnaire** of the study "**Effects of Exhibit Text Presentation Types on Visual Attention Patterns and Cognitive Load in Digital Museums**". I understand that I will receive a signed copy of this form.

___________________	____________________	___________________
Participant's Name (Print)	Participant's Signature	Date

By marking my initials here, _______, I agree to allow the researchers to contact me at a later time if they would like me to clarify any information.

Person obtaining consent:

I have discussed this study in detail with the participant. I believe the participant understands what is involved in this study.

_______________________	______________________	________________
Researcher's Name (Print)	Researcher's Signature	Date

If you have any questions regarding the ethical conduct of this study, you may contact the principal investigator Tel.: +86-135-27223135, Email: summerlion@kookmin.ac.kr.
