# Supplementary material for: Effects of Exhibit Text Presentation Types on Visual Attention Patterns and Cognitive Load in Digital Museums: An Eye-Tracking Study
Source: J Eye Mov Res. 2026 Aug 3;19(4):84. doi: 10.3390/jemr19040084 (PMC13514559; doi:10.3390/jemr19040084)
Supplement: Supplementary file 1 [file jemr-19-00084-s001.zip › File S4.pdf]

File S4. Semi-structured Interview Three-level Framework

Process	Dimension	Question
Warm-up	Reflecting on past experiences	Q0-1. Do you usually visit online museums or digital art exhibitions? Could you give a few examples?<br>Q0-2. Do you typically browse online museums or digital exhibitions using a computer or a mobile phone?<br>Q0-3. In your impression, what is the biggest difference between visiting an online exhibition and visiting one offline?
Task	Please rank the three presentation types from **easy to difficult** based on your experience when reading the textual information of the artwork. Please also explain the reasons for your ranking.	
Key Questions<br><br>1.Overall Experience<br><br>2.Text Presentation Format<br><br>3.Expectations	Interactive Experience	Q1. When you enter an online exhibition, what makes you want to continue viewing the exhibition? (What attracts you?)<br>Q2. What difficulties did you encounter when browsing these digital exhibits?<br>Q3. During operation or browsing, do you think the system response or guidance was sufficient?<br>Q4. Compared with offline exhibitions, in what aspects do you think online exhibitions are more convenient or more limited?
	Visual aspect	Q5. Among the different text presentation types, which one felt most comfortable for reading? Why?<br>Q6. When the explanatory text appeared or changed, did it interrupt your focus on the artwork itself?
	Information Comprehension	Q7. Which presentation method helped you understand the artwork best? Why?<br>Q8. During reading, did you feel that the content was too much, too fast, or too complex? Was this more obvious in a particular presentation format?<br>Q9. In certain presentation formats, were you more likely to become distracted or ignore the text?<br>Q10. Overall, which exhibit information layout did you prefer? Why?<br>(Please consider aspects such as visual aesthetics, reading efficiency, and level of understanding.)
	Expectations	Q11. If future online exhibitions adopt a new interactive text format, what would your ideal reading experience look like?

		Q12. If you could give suggestions to digital museum designers, what improvements would you like them to make regarding explanatory text?<br>Q13. What aspects do you think digital exhibitions should emphasize more (e.g., artistic value, educational value, interactivity, convenience)? What would your ideal viewing experience be like?
