# Supplementary material for: Effects of Exhibit Text Presentation Types on Visual Attention Patterns and Cognitive Load in Digital Museums: An Eye-Tracking Study
Source: J Eye Mov Res. 2026 Aug 3;19(4):84. doi: 10.3390/jemr19040084 (PMC13514559; doi:10.3390/jemr19040084)
Supplement: Supplementary file 1 [file jemr-19-00084-s001.zip › File S5.pdf]

File S5

It presents the results of an additional exploratory 3 × 2 linear mixed model analysis. This analysis was conducted to examine whether viewpoint influenced the eye-tracking results before averaging the front-view and side-view data for the main analysis. In the model, presentation condition (EN, OB, and US), viewpoint (front and side), and interaction were included as fixed effects, while participant was included as a random intercept.

Table E1. Results of the supplementary 3 × 2 linear mixed model analysis

Variable	Condition	Viewpoint	Condition × Viewpoint
Blink rate	$F(2,185) = 4.166$, $p = 0.017$	$F(1,185) = 0.039$, $p = 0.843$	$F(2,185) = 1.273$, $p = 0.282$
Text AOI fixation count *	$F(2,185) = 6.653$, $p = 0.002$	$F(1,185) = 0.643$, $p = 0.423$	$F(2,185) = 3.986$, $p = 0.020$
Text AOI fixation duration	$F(2,185) = 6.238$, $p = 0.002$	$F(1,185) = 0.314$, $p = 0.576$	$F(2,185) = 1.070$, $p = 0.345$
Text AOI Mean Fixation duration	$F(2,185) = 0.550$, $p = 0.578$	$F(1,185) = 0.013$, $p = 0.910$	$F(2,185) = 3.351$, $p = 0.137$
Image AOI ↔ Text AOI transition count	$F(2,185) = 5.816$, $p = 0.004$	$F(1,185) = 1.496$, $p = 0.223$	$F(2,185) = 2.471$, $p = 0.087$

* AOI = area of interest

Table E2. Summary of significant effects in the supplementary 3 × 2 analysis

**Test component**	**Result**
Main effect of Condition	Significant in 4 of the 5 measures
Main effect of Viewpoint	Not significant in all 5 measures
Condition × Viewpoint interaction effect	Significant in 1 of the 5 measures

Given the self-paced nature of the viewing task, overall viewing time was additionally examined as a potential confounding factor. The linear mixed-effects model showed no significant effect of presentation condition on overall viewing time, ($F(2, 74) = 1.253$, $p = 0.292$, $\eta_p^2 = 0.033$), indicating that the overall time spent completing the task was broadly comparable across the three conditions.

Table E3. Linear Mixed-Effects Model Results for Overall Viewing Time

**Outcome**	**df1**	**df2**	**F**	**p value**	**Partial** η_p^2
Overall viewing time	2	74	1.253	0.292	0.033
